# Online Coaching of Emotion-Regulation Strategies for Parents: Efficacy of the Online Rational Positive Parenting Program and Attention Bias Modification Procedures

**DOI:** 10.3389/fpsyg.2017.00500

**Published:** 2017-04-04

**Authors:** Oana A. David, David Capris, Alexandra Jarda

**Affiliations:** Department of Clinical Psychology and Psychotherapy, Babes-Bolyai UniversityCluj-Napoca, Romania

**Keywords:** parent attention bias modification, the rational positive parenting program, positive parenting, parent reappraisal, acceptance parenting

## Abstract

Parenting programs are currently treatment of choice for behavioral disorders in children and one of their main components is reducing the negativity bias in the child–parent dyad. The Rational Positive Parenting Program (rPPP) is a program with a special focus on parent emotion-regulation functional reappraisal strategies, which has recently received consistent support for reducing child externalizing and internalizing disorders. In the last years, online interventions were proliferated and the Attention Bias Modification (ABM) becoming a promising implicit therapeutic intervention based on attention deployment emotion-regulation strategy, or adjunctive module to usual treatments, with results in multiple domains, varying from pain to self-esteem and emotional disorders (e.g., anxiety). We conducted two studies to investigate (1) the efficacy of the ABM procedures applied to parents and (2) the efficacy of the online version of the rPPP augmented with an ABM module. A total of 42 parents of children aged 2–12 years old participated in the first study, being allocated either to the ABM training or wait-list. Positive results were reported by the parents participating in the ABM group for own distress, satisfaction, positive interactions with the child, and child’s strengths. In the second study, 53 parents and their children were allocated either in the rPPP group or in the rPPP + ABM group. Results show that ABM training can boost the effects of the rPPP on the strengths of children reported by the parents after the intervention. Findings are discussed in the light of limited research on using online tools for coaching effective emotion-regulation strategies for parents.

## Introduction

Attentional distraction is considered an antecedent emotion-regulation strategy (Gross, 1998) that involves shifting attention from one aspect of a situation to another one, or shifting ones’ attention away from the situation altogether. It is well known that in the case of children with externalizing disorders, a negative interaction cycle arises, which is affecting the child–parent relationship and is thus contributing to maintaining children’s problems (Barkley, 1997). Parents get biased to pay attention preponderantly to the negative behaviors of their children, which in turn impacts negatively on the parent–child relationship and their parenting style. Parenting programs (cognitive-behavioral) are considered treatment of choice for child disruptive behavior, receiving considerable support (Lundahl et al., 2006). More recently, parent emotion-regulation has started to be addressed in such parenting programs and this was shown (David et al., 2014b) to augment their effects on both parent and child outcomes.

Starting from the promising results regarding the effects of computerized psychological treatments, during the past 15 years parenting programs have started to be adapted for being implemented online (Feil et al., 2008; Enebrink et al., 2012), in order to make them more accessible to the parents in need. A recent review shows (see Nieuwmboer et al., 2013) robust positive effects of such parenting programs, similar to those of the “face to face” programs. The online format of such programs, however, opens unlimited opportunities for innovative procedures which could improve their outcomes.

Newly developed interventions aimed at modifying negative attentional biases (i.e., attention preferentially allocated to negative, disliked, or threat like stimuli) by training implicit associations have been recently suggested (David and Podina, 2014) as offering important strategies in fostering positive parenting. Training of implicit attentional associations from negative stimuli toward to positive or neutral stimuli has received much interest with the introduction of the Attention Bias Modification (ABM) training. Negative attention biases have been etiologically linked (Hakamata et al., 2010; Hallion and Ruscio, 2011; Bar-Haim et al., 2007; Eldar et al., 2012; Waters et al., 2013) to various mental health issues in both adults and children. In parents, current negative attention biases toward their children’s negative behavior can impede them in implementing the strategies learned during a parenting program. In this context, we consider that ABM bares the promise of helping parents to allocate attentional resources toward prospective positive responses in children, which in turn could increase the efficacy of the parenting programs.

Although the ABM paradigm has not been extended to the parenting field up until now, there is great potential for this domain. Our aim was to investigate the efficacy of the ABM as an online intervention based on attention deployment emotion-regulation mechanisms delivered to parents on various parenting and child outcomes; then, in a second step, we aimed to investigate the additive effect of including an ABM module within an online parenting program curriculum.

## Study 1

ABM is based on the emotion-regulation attentional deployment strategy, considered a new intervention within the cognitive-behavioral therapy framework, that has been documented to have promising clinical effects in both youth and adult population (Bar-Haim, 2010; Hakamata et al., 2010; Hallion and Ruscio, 2011; Eldar et al., 2012; Waters et al., 2013), regarding anxiety and emotional related issues. The initial forms of ABM trained attention to neutral benign stimuli and was found to have mixed results. More recent procedures were developed, however, as alternative that train attention toward positive or rewarding stimuli (Dandeneau et al., 2007).

When involved in the ABM training to positive stimuli [e.g., visual search task training (VSTT) paradigm; Dandeneau and Baldwin, 2004; Dandeneau et al., 2007], participants are instructed to preferentially process happy faces to the expense of the angry ones. Although it represents a work in progress (due to mixed findings and unknown mechanisms of change), the ABM could be an essential component for parenting programs.

It is known that negative cognitive biases in parents can affect their parenting skills (Podina et al., 2013; David et al., 2014a), and at the same time parental cognitive biases can facilitate an intergenerational transmission of mental health issues. Thus, the present paper aims to extend the existing ABM paradigm, namely to investigate for the first time its efficacy in boosting parenting skills. More specifically, we intend to use faces of children to reduce negative biases in parents. The novelty of this approach is that the beneficiaries of the ABM training will not be the user, meaning the parents, but their children. Given the previous arguments, such a procedure would be useful especially in the context in which parents’ negative biases regarding their children behaviors interferes with a good parent–child relationship.

The current study aims at investigating the efficacy of an ABM intervention delivered by itself in reducing parents’ negative interactions with children, distress, and improving their parenting, self-efficacy, satisfaction, and child externalizing and internalizing reported symptoms. We will compare in a superiority trial design the efficacy of the ABM intervention for parents with a wait-list (WL) group. Thus, we expect that the parents participating in the ABM intervention will report better outcomes compared to the WL condition.

### Methods

#### Participants

A total of 42 parents participated in this study, 36 mothers and 6 fathers of children aged 2–12 years old (*M* = 5.93, *SD* = 2.59). Their age range varied between 24 and 43 years old, with a mean age of 32.96 (*SD* = 5.31). Forty-one percent of the parents included in the study had only one child, while 25% had two children, and 2% had three children. They were asked to report regarding the behavior on one child, and 25 of the children selected were boys, while the rest were girls. Ninety-two percent of the parents were married, one parent was in an unofficial relationship, and three were divorced. Most of the parents had bachelor (33.8%) and college education (45.6%), while most of them (89.7%) had urban residence; 41 had a socio-economic background above the minimum wage of the country, while 22 were earning above the mean medium salary of the country.

#### The ABM Procedure

We chose the standard ABM procedure (Amir et al., 2009; Bar-Haim, 2010; Waters et al., 2013) using the faces of children. The parent ABM task aims to redirect the attention of parents from angry faces of children to happy faces of children, for prevention purposes.

We used a modified version of the dot-probe task (MacLeod et al., 1986), and developed our training using the Inquisit 3 version of the software Milliseconds, which is used on a large scale by researchers (e.g., Thoern et al., 2016). During the probe, participants view pictures with faces of children. The faces represent negative emotions (e.g., anger), positive (e.g., joy), or neutral emotions, with two types of emotions being presented at once. After being presented with the pair of pictures, participants are asked to press the E or F keyboards that appear in their places. During the ABM training, similar with the probe task, the letters E and F follow only pictures presenting faces of children that convey positive emotions in order to train orientation of implicit attention from negative stimuli toward the positive ones. The underlying mechanism is classical conditioning, and thus the focus of attention is associated with positive stimuli.

The training was delivered over the course of 1 week, with five online sessions, as recent studies indicate that even a few (one to two) sessions are sufficient to train a positive bias. We chose to use more sessions as we wanted to boost the learning of positive stimuli and provide the opportunity for the training to be delivered in multiple contexts (e.g., at home, at work). The chosen stimulus set consisted of angry and happy faces of children selected from the NIMH-ChEFS data base (Egger et al., 2011). Each training session lasted approximately 15 min daily and thus 60 min per week. Participants had a first contact with a clinical psychologist, and then they maintained contact via email and phone, being provided with information regarding their status and following steps. Parents were instructed not to take breaks during the training session and the completion of their training was monitored online daily.

#### Measures

Parents completed questionnaires regarding their child strengths and difficulties and their own parenting practices, stress, and attitudes. The measures used were chosen based on their relevance for outcomes considered in the study, their psychometric properties and large use in the parenting or emotion-regulation field.

##### The Strengths and Difficulties Questionnaire (SDQ; Goodman, 1997)

SDQ is an instrument measuring behavioral and emotional problems in children and adolescents. The instrument produces scores for five subscales: conduct problems, hyperactivity, emotional problems, peer problems, and prosocial behavior. Each subscale consists of five items (Graf et al., 2014). The scale has demonstrated adequate psychometric properties on national population (Colvert et al., 2008) and in our sample (α = 0.82). We used the standard SDQ in the study.

##### The Parent Behavior Inventory (PBI; Lovejoy et al., 1999)

The PBI is a 20-item self-report scale. Parents rate the frequency of various parenting behaviors (e.g., hugging, teaching new things) using a 6-point Likert scale (0, never true to 5, almost always true). This instrument includes two factors: supportive/engaged parenting and hostile/coercive parenting. The total scores for the supportive/engaged parenting subscale range from 0 to 50, higher scores representing higher levels of supportive/engaged parenting. The total scores for the hostile/coercive scale range from 0 to 35, with higher scores representing higher levels of hostile/coercive parenting. The scale has adequate psychometric properties (α = 0.82, Bruce et al., 2006; and α = 0.72 in our sample).

##### The Parent Stress Scale (PSS; Berry and Jones, 1995)

The PSS is an 18-items self-report scale which measures the positive aspects of parenthood (emotional benefits, self-enrichment, personal development) as well as negative indicators (demands on resources, restrictions, and opportunity costs). The final score is given by the sum of items, higher scores indicating greater stress. This scale demonstrated good internal reliability on the national population included (Cronbach’s α = 0.85; David et al., 2014a used on national population) and in our sample (α = 0.82).

##### The Parenting Sense of Competence Scale (Gibaud-Wallston and Wandersman, 1978)

The Parenting Sense of Competence (PSOC) Scale is a 16 items questionnaire which measures parents’ views of their competence as parents. This questionnaire includes two subscales: satisfaction with their parenting role and feelings of efficacy as a parent. Satisfaction subscale examines parents’ anxiety, frustration, and motivation, while the efficacy subscales examines parents’ competence, capability levels, and problem-solving abilities in their parenting role. High scores suggest a higher level of satisfaction, while low self-efficacy scores were correlated with behavioral problems in children. Psychometric proprieties of the total score and the subscales are adequate (alpha Cronbach’s for the total score is α = 0.71; Johnston and Mash, 1989; for national population: Gavita et al., 2014, and α = 0.85 in our sample) showing that it can be used in research.

#### Mean Positive and Negative Interactions

Parents were asked to estimate the weekly number of positive and negative interactions which they had with their child, keeping in mind the number of daily interactions. The examples of positive interactions taken into account by parents were the following: praise, physical affection, laughter, performing an act requested by the child, positive gestures or any other positive interaction. The examples of negative interactions were the following: yelling, negative physical contact (pulling, pushing, slapping), not performing an act requested by the child, negative gestures, repeating a request insistently or any other negative interaction.

#### Procedure

Parents were recruited from the kindergartens and schools where their children were enrolled. Parents were randomly distributed among the experimental group and the waitlist. They filled the baseline assessment (pre-test) and the same questionnaires after 1 week (post-test). Participants signed an online informed consent about participating in the study and received detailed information about study procedure. This study was approved by the Institutional Review Board of the Babes-Bolyai University (GTC-34060/2013).

#### Data Analysis

We used repeated measures ANOVA with Time (pre–post) as a within-subject factor and Group (ABM, WL) as a between subjects variable, for each of the outcomes. Pre-test data was lost in the case of the SDQ and PBI questionnaires due to an error in the online platform and thus only univariate analyses of the post-test data were possible for these measures. We use intent to treat analyses, imputing the missing data at post-test in order to minimize the risk for type 1 error.

### Results

**FIGURE 1:**
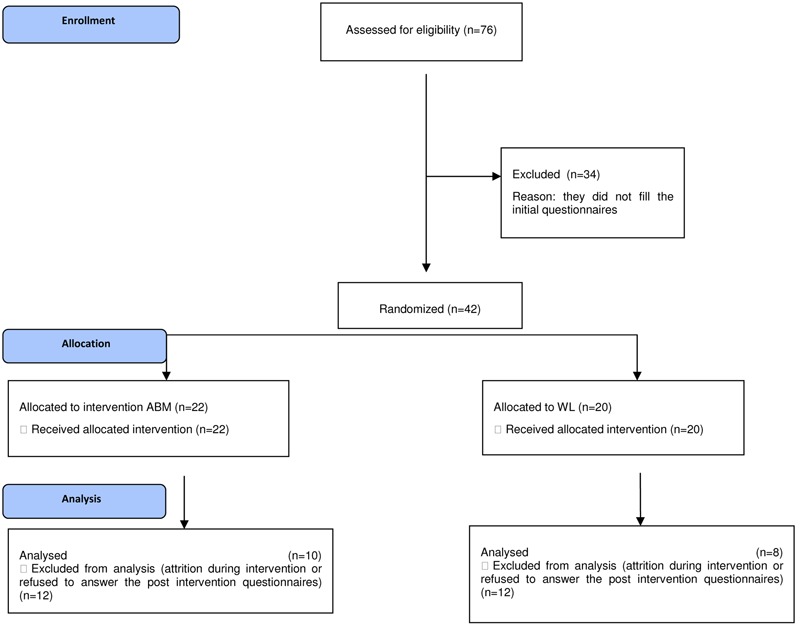
**Consort flow diagram of the phases of the ABM versus WL trial**.

No significant differences were found in terms of the demographics among the groups (*p*s > 0.5). The phases of the trial are presented in **Figure 1**. Due to high drop-out rates registered (54% in the ABM group and 40% in the WL), we analyzed potential reasons, and found that drop-outs had significantly lower educational level [χ^2^(4) = 0.38, *p* = 0.034], and monthly income [χ^2^(5) = 15.69, *p* = 0.008].

#### Descriptive Analyses

Means and standard deviations for the outcomes in each group are presented in **Table 1**.

**Table 1:** Means and standard deviations for the outcomes in each of the groups.

Group	Time	ABM		WL		Total	
		*M*	*SD*	*M*	*SD*	*M*	*SD*
Parental distress	Pre	34.82	4.79	31.00	7.62	32.37	6.24
	Post	34.73	6.01	35.22	11.36	33.58	8.38
PSOC—self-efficacy	Pre	32.82	4.14	35.20	6.49	34.20	5.23
	Post	33.45	5.05	33.95	5.89	34.20	5.26
PSOC—parent satisfaction	Pre	40.86	4.31	41.45	8.12	41.80	6.04
	Post	40.68	5.58	38.95	8.59	40.76	6.60
PSOC—self-esteem	Pre	73.68	6.47	76.65	13.59	76.00	9.73
	Post	74.18	8.10	72.90	13.46	75.14	10.12
Positive interactions	Post	7.38	4.09	5.75	1.69	6.39	3.57
Negative interactions	Post	5.10	4.00	3.31	0.79	4.24	3.60
PBI-supportive parenting	Post	43.88	7.08	38.20	6.94	41.40	7.43
PBI coercive parenting	Post	19.88	7.64	12.80	3.79	16.36	6.64
SDQ strengths	Post	12.40	1.96	4.50	3.03	7.93	4.19
SDQ difficulties	Post	35.30	3.43	18.80	5.49	26.15	8.85

#### Inferential Analyses

For parental distress, multivariate tests show a significant main effect of Time, *F*(1,47) = 9.73, *p* = 0.004, χ^2^ = 0.166, a significant main interaction effect of Time × Group, *F*(1,47) = 13.32, *p* = 0.001, $\eta_{p}^{2}$ = 0.221, and a non-significant main effect of Group (*p* > 0.05). No significant differences were obtained pre–post for the experimental group (*p* > 0.05), while the WL group reported increases in stress [*t*(21) = 3.69, *p* = 0.001].

For parent satisfaction, multivariate tests show a significant main effect of Time, *F*(1,49) = 6.59, *p* = 0.013, $\eta_{p}^{2}$ = 0.11, a significant main interaction effect of Time × Group, *F*(1,49) = 7.47, *p* = 0.009, $\eta_{p}^{2}$ = 0.1032, and a non-significant main effect of Group (*p* > 0.05). Pre–post significant reductions in parent satisfaction were registered only in the WL condition [*t*(23) = 4.38, *p* < 0.001].

For parent self-esteem, multivariate tests show a marginally significant effect of Time, *F*(1,49) = 3.10, *p* = 0.084, $\eta_{p}^{2}$ = 0.06, a significant main interaction effect of Time × Group, *F*(2,56) = 5.19, *p* = 0.003, $\eta_{p}^{2}$ = 0.17, and a non-significant main effect of Group (*p* > 0.05). No significant differences were obtained pre–post for the experimental group (*p* > 0.05), while the WL group reported increases in self-esteem [*t*(23) = 3.62, *p* = 0.001].

For parental self-efficacy, multivariate tests show a significant main interaction effect of Time × Group, *F*(1,49) = 10.21, *p* = 0.002, $\eta_{p}^{2}$ = 0.172, and a non-significant main effect of Time or Group (*p* > 0.05). No significant differences were obtained pre–post for the experimental group (*p* > 0.05), while the WL group reported pre–post significant increases in self-efficacy [*t*(23) = 2.51, *p* = 0.019].

In terms of the child and parenting at post-test, univariate analyses showed that parents in the ABM group reported significantly more daily positive interactions *F*(1,22) = 6.29, *p* = 0.012, $\eta_{p}^{2}$ = 0.059, more child strengths *F*(1,22) = 6.29, *p* = 0.005, $\eta_{p}^{2}$ = 0.70, and more supportive parenting *F*(1,20) = 3.25, *p* = 0.086 (not statistically significant), $\eta_{p}^{2}$ = 0.14, compared to the WL group. No significant differences were obtained in terms of daily negative interactions, child difficulties or coercive parenting between groups at post-test.

### Discussion

We investigated the efficacy of a 1 week ABM procedure in parents compared to a wait-list. Parents participating in the intervention group registered significantly better outcomes compared to the wait-list, in terms of parent distress, satisfaction, and child strengths, as reported by the parents. However, we cannot exclude the fact that the effects could be explained by the wait-list condition changes rather than the significant changes in the ABM group. There are findings in the literature showing that patients on the wait list can improve during the trial (Hesser et al., 2011) due to factors such as the therapeutic contact or expectations, and thus such an effect could have arisen in our study. Future studies would need to also investigate potential mechanisms involved, such as hope or expectancies. Since it was suggested (Bar-Haim, 2010) that the ABM procedure might augment the effects of the standard treatments, further research should investigate the efficacy of such programs. An important limitation of the study is the missing baseline data regarding child behavior and parenting.

## Study 2

One of the key components of any parenting program is to train parents for detecting and reinforcing positive (i.e., adaptive) behaviors, attitudes, and emotions in children in order to increase their frequency in the expense of the maladaptive ones. Although numerous advantages can be derived from positive reinforcement of the adaptive responses in children and obvious focus of the parenting programs of reinforcing an adaptive response when detected, the actual detection and reinforcement to positive responses in children remains an issue of concern. Most parenting programs fail to provide parents with training on how to spot and recognize the targeted cluster of positive responses (behavior, attitudes, and cognitions) in their children. Such training would be most important for a specific group of parents, the ones with children who display disruptive behaviors.

Some online parenting programs curricula have been recently investigated (Feil et al., 2008; Enebrink et al., 2012) in terms of their effects for child and parent outcomes. A meta-analytic review of these studies (see Nieuwmboer et al., 2013) shows medium effect sizes of the online parenting programs across both parent and child outcomes. The Rational Positive Parenting Program (rPPP; Gavita et al., 2013; David and DiGiuseppe, 2015) is a cognitive-behavioral program which has recently received support for both its full-length format (David et al., 2014b) and short-lenght format (Gavita et al., 2012), face to face and self-help (Gaviţa and Călin, 2013). Moreover, this program is emphasizing the focus on parent emotion-regulation skills and has documented (David, 2014) the importance of these improvements as mechanisms for child outcomes.

This study aims to investigate the efficacy on the online version of the rPPP compared with its version augmented with the ABM procedure in improving parent and child outcomes. We hypothesize that the parents participating in the rPPP + ABM intervention will report better outcomes compared to the parents participating in the rPPP intervention.

### Methods

#### Participants

A total of 53 parents participated in this study, 48 mothers and 5 fathers. Parents were aged 24–57 years old (*M* = 35.97, *SD* = 5.25). 60.4% the parents (30) had only one child, while 33.2% had two and 6.9% had three children. Parents chose the child to which they referred while responding to the questionnaires. Children referred to were aged 2–12, with a mean age of 6.45 (*SD* = 3.34), 30 of them being boys and 23 girls. 79.2% of the parents were married and 1.9% declared themselves to be unmarried, while 5.7% were in an unofficial relationship, 7.5% were divorced, and 5.7% were separated. 92.5% of the parents had earnings above the minimum country wage, while 43.4% of them earned above the mean country wage. In terms of the educational status, 71.7% had graduate and higher level of education. Forty-three of the parents lived in the city, while the rest lived in the rural areas.

#### Measures

The SDQ, PBI, PSS, PSOC, and positive/negative daily interactions were measured using the same measures presented above. The Parent Rational and Irrational Beliefs Scale (P-RIBS; Gavita et al., 2011a) was used to measure irrational cognitions in parents.

##### The P-RIBS (Gavita et al., 2011a)

P-RIBS is measuring rational and irrational beliefs conceptualized as opposite constructs, but not at opposite poles. The scale contains 20 items, constructed to reflect the four irrational beliefs (demandingness, awfulizing, low frustration tolerance, and global evaluation) and four rational beliefs (preferences/flexibility, negative evaluations, frustration tolerance, and unconditional acceptance). The first part of the scale measures the child behavior and the second, the parent behavior. The total score of the scale is given by the sum of items, with rational items scored in a reversed way. Internal consistencies of the scale showed adequate psychometric properties on national population (Gavita et al., 2011a; α = 0.85 in our sample).

#### Procedure

**FIGURE 2:**
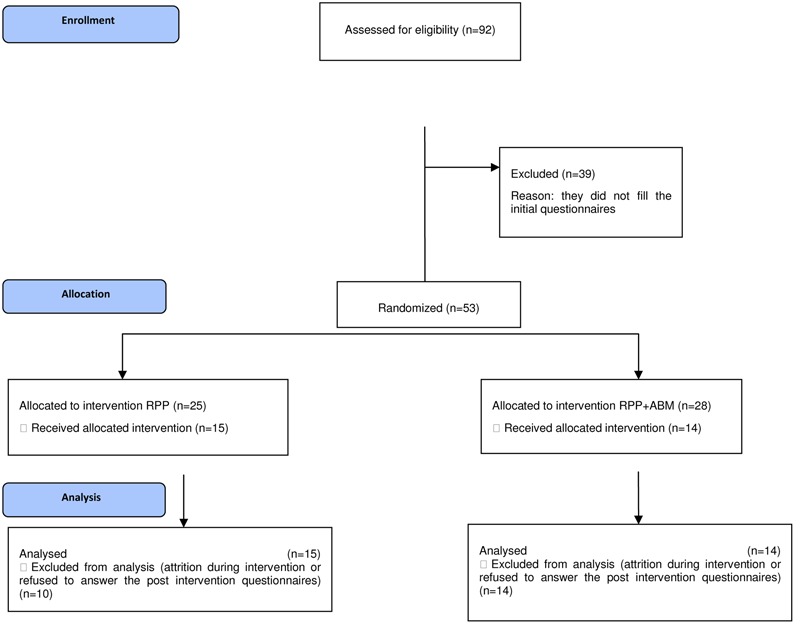
**Consort flow diagram of the phases of the rPPP versus rPPP + ABM trial**.

All parents were recruited from the kindergartens and schools where their children were enrolled. Parents were randomly allocated 25 in the rPPP group and 28 in the rPPP + ABM group, as presented in **Figure 2**. Participants signed an online informed consent form prior to being included in the study, and were informed about the main purposes of the study, about the confidentiality of the data, risks and the possibility to withdraw from the study at any time. Participants had a first face to face contact with the clinical psychologist delivering the program, after which they were provided with information regarding their status, following steps, and reminders via the email and phone texts.

##### The Rational Positive Parenting Program

The online version of the rPPP consists of eight modules (David and DiGiuseppe, 2015). Participants received via e-mail web links in order to access each module, with a constant rhythm of tow modules per week. At 2 days after receiving the e-mail with a module, participants who did not access it, were sent a reminder via e-mail and a text message by phone. If they did not reply, the next day they received a phone call. The first module of the program offers a rationale for the program, sets goals for change, and educates about the behavioral problems of children and their causes. Parents are asked to monitor their child’s behavior using a chart based on functional analysis. The next two modules aim to teach participants emotional regulation strategies based on the cognitive-behavioral theory. Parents are taught the ABC model and how to identify the cognitions that cause their emotions toward their children, and how to tackle these cognitions. A difference between dysfunctional and non-dysfunctional negative emotions is made and homework is given (to fill cognitive ABC forms and rational statements). After participants learn to manage their parental stress, the next five modules aim to teach them positive parenting skills and strategies needed for child behavior management (e.g., functional analysis of the behavior, efficient rewarding, ignoring and distraction, family rules, prompting, efficient usage of consequences, time-out, reflective listening). The last of the modules is used to help parents to establish a prevention plan, teach them problem solving skills, and summarize what has been learned in the program.

##### The Rational Positive Parenting Program plus Attention Bias Modification (rPPP + ABM)

The rPPP + ABM consisted, besides the standard rPPP, of the ABM component described in Study 1, delivered in the so called 7th+ session, with the purpose of reducing the cognitive bias of parents toward the negative stimuli (angry faces of children) and training it toward the positive emotion faces. Participants were asked to follow the ABM training at least once a day for 1 week.

#### Data Analysis

We used repeated measures ANOVA with Time (pre–post) as a within-subject factor and Group (rPPP, rPPP + ABM) as a between subjects variable, for each of the outcomes. Pre-test data could not be used the case of the SDQ and PBI questionnaires due to the online platform error and thus only univariate analyses of the post-test data was possible for these measures.

### Results

No differences were found between groups in terms of the demographic variables. In terms of the attrition rate, 10 parents of the rPPP group dropped out, while 14 of the rPPP + ABM dropped until the end of the trial. The phases of the trial are presented in **Figure 2**. We used intent to treat analyses in order to minimize the risk for type 1 error. Due to the high drop-out rate, we analyzed the differences between completers and dropouts in terms of their demographic characteristics and found that significantly more fathers dropped-out [χ^2^(1) = 0.0368, *p* = 0.032].

#### Descriptive Analyses

**Table 2** presents the means, standard deviations, and effect sizes of the pre–post changes (Cohen’s *d*) for the outcomes in each of the groups.

**Table 2:** Means, standard deviations, and effect sizes (Cohen’s *d*) for the outcomes in each of the groups.

Variable	Group Time/coefficient	rPPP			rPPP + ABM			*d*
		*M*	*N*	*SD*	*M*	*N*	*SD*	
Parental distress	Pre	31.40	25	7.35	34.61	28	8.68	–
	Post	28.64	25	7.86	32.50	28	9.10	-0.49
Parent irrational beliefs	Pre	53.40	25	6.38	55.29	28	8.50	–
	Post	51.64	25	5.92	52.85	28	10.43	0.14
Parental self-efficacy	Pre	34.12	25	6.23	33.07	28	4.89	–
	Post	37.32	25	6.34	36.35	28	6.48	-0.15
Parental satisfaction	Pre	41.52	25	7.98	42.14	28	6.71	–
	Post	43.96	25	7.55	44.78	28	8.03	0.10
Parental self-esteem	Pre	75.64	25	12.82	75.21	28	9.43	–
	Post	81.28	25	13.16	79.78	28	12.32	-0.11
Supportive parenting	Post	47.27	11	3.00	46.50	10	2.64	-0.27
Coercive parenting	Post	15.45	11	8.35	14.80	10	9.54	-0.07
Child strengths	Post	10.18	11	4.17	13.27	11	1.56	0.98
Child difficulties	Post	16.27	11	7.20	16.82	11	5.64	0.08
Daily positive interactions	Post	6.50	24	1.93	6.57	28	3.01	0.02
Daily negative interactions	Post	3.21	24	2.30	3.79	28	2.35	0.24

#### Inferential Analysis

For parental distress, multivariate tests show a significant main effect of Time, *F*(1,51) = 11.73, *p* = 0.001, $\eta_{p}^{2}$ = 0.187, a non-significant interaction effect of Time × Group, and main effect of Group (*p*s > 0.05). We obtained significantly lower scores in parent’s distress from pre to post intervention for both the rPPP group [*t*(24) = 3.22, *p* = 0.004], and marginally for the rPPP + ABM group [*t*(27) = 1.90, *p* = 0.067].

For parent irrational cognitions, multivariate tests show a significant main effect of Time, *F*(1,51) = 10.88, *p* = 0.002, $\eta_{p}^{2}$ = 0.176, a non-significant interaction effect of Time × Group, and main effect of Group (*p*s > 0.05). Significant pre–post reductions in irrational thinking were obtained for the rPPP group [*t*(24) = 2.21, *p* = 0.036] and for the rPPP + ABM group [*t*(27) = 2.51, *p* = 0.018].

In terms of parent satisfaction, multivariate tests show a significant main effect of Time, *F*(1,51) = 11.005, *p* = 0.002, $\eta_{p}^{2}$ = 0.177, and a non-significant interaction effect of Time × Group, or main effect of Group (*p*s > 0.05). Significant pre–post improvements in parents’ satisfaction were obtained for both the rPPP group [*t*(24) = -3.27, *p* = 0.003], and for the rPPP + ABM group [*t*(27) = -2.24, *p* = 0.034].

For parent self-esteem, multivariate tests show only a significant main effect of Time, *F*(2,51) = 18.18, *p* < 0.001, $\eta_{p}^{2}$ = 0.263, and a non-significant interaction effect of Time × Group or main effect of Group (*p* > 0.05). Significant pre–post changes in parents’ self-esteem were obtained for the rPPP group [*t*(24) = -3.65, *p* = 0.001], and for the rPPP + ABM group [*t*(27) = 2.54, *p* = 0.017].

For parental self-efficacy, multivariate tests show a significant main effect of Group, *F*(2,51) = 20.99, *p* < 0.001, $\eta_{p}^{2}$ = 0.292, a non-significant main interaction effect of Time × Group or main effect of Group (*p*s < 0.05). Significant pre–post improvements in parents’ self-efficacy were obtained for the rPPP group [*t*(24) = -3.13, *p* = 0.004], and for the rPPP + ABM group [*t*(27) = -1.27, *p* = 0.002].

In terms of the daily positive or negative interactions reported at post-test, we did not obtain significant differences between the groups (*p*s > 0.05). For coercive parenting and supportive parenting we did not found significant differences between the groups (*p*s > 0.05). No differences were obtained regarding the difficulties experienced after the programs (*p*s > 0.05). In terms of the strengths of children reported by parents after the interventions, rPPP + ABM worked better *F*(1,19) = 5.31, *p* = 0.032, compared with the rPPP group.

### Discussion

The present study investigated the efficacy of the online version of the rPPP as standalone versus its augmented version with ABM. Results show that both versions of the rPPP proved to be effective in improving the emotional and attitudes outcomes, with high effect sizes. The magnitude of changes obtained following participation in the online sPPP, both in terms of parent-related outcomes (parenting, self-efficacy, self-esteem, distress) and child-related outcomes (interactions), is in line with findings in the literature reporting comparable effects of the online parenting programs to those delivered face to face (see Nieuwmboer et al., 2013). No significant differences were obtained between the effects of the programs, as hypothesized, regarding parent distress, parent self-efficacy, satisfaction, self-esteem, parenting practices. The additive effects of the rPPP augmented with the ABM was found to be significantly higher only in the case of parent reported child strengths, with a high effect size.

This is the first study investigating the additive effects of an ABM enhanced online parenting program, namely the rPPP. The study offers important preliminary data regarding the effects of integrating the ABM module within the well-researched parenting programs. Considering the accessible format of the online parenting programs, the ABM computer-based format is especially suitable. However, more studies are necessary for documenting the cost-benefits balance, considering the high attrition rate of a longer intervention and the little support for augmented changes. Moreover, future studies should use a componential analysis, while incorporating intermediate measures. An important limitation of our study is the lack of the baseline measure for child behavior and parenting, the small sample size and high attrition rate.

## General Discussion and Conclusion

Training parents for detecting positive responses in children can be considered central to current parenting programs, in that it can assist parents in effortlessly detecting positive behaviors in their children, and giving them the chance to reinforcing them. We proposed (see also David and Podina, 2014) that the ABM procedures can be especially suited for online delivered parenting programs. Thus, we integrated the ABM training at the end of the rPPP but found no benefits on most of the outcomes compared with the parenting program alone. However, this could be due to the fact that its integration might be most useful in the initial phases of the parenting intervention, for helping parents in detecting positive responses from their children. The ABM procedures could be also integrated both throughout the parenting programs, and during additional booster sessions. Additionally, ABM could be an especially useful tool for parents with a negative cognitive pattern (e.g., distressed or depressed mothers). Since a positive attentional bias can transfer to other processing levels, such as interpretation or memory bias, boosting emotion-regulation could bring important effects on optimal parent–child interaction. In fact, it might be that parents of children with externalizing disorders would profit from the addition of the ABM training to standard parenting tools. For these parents ABM could offer special coaching in detecting positive behaviors in their children, due to their pre-existing biases to primarily detect the negative ones.

An important limitation of both studies is that we used a classical ABM paradigm as opposed to the VSTT paradigm (Dandeneau and Baldwin, 2004) which showed positive results and offers the gaming advantage. David and Podina (2014) developed a parent VSTT involving a game-based search for a happy child face embedded in a matrix of angry faces. The nature of the task and its interactive features make it and attractive and promising tool for boosting self-esteem, based on the previous findings regarding improvement of self-referential processing. Future studies should document its effectiveness in training parents’ attention toward positive child cues.

We believe that online delivered parenting tools with implicit components, like the ABM training, are in support of their aims. The online format can make the intervention more easily accessible and bring cost-effectiveness benefits compared to the standard parenting programs Moreover, the implicit component is could ease the work of parents, by automatizing the negative bias correction. Future studies will need to document ways to minimize the high dropout rates registered by us and reported in the internet-based and parenting programs literature (Gavita et al., 2011b). Since another limitation of present studies is the lack of follow-up assessment, future studies will need to investigate the long-term efficacy of such parenting interventions. Also, future studies will need to investigate comparative efficacy of the online parenting program with an active control group, such as parental support.

Building on studies indicating that cognitions are key determinants of parenting skills (Gavita, 2011), the rPPP brings a new spin to available programs, in that it focuses on components related to boosting emotion-regulation of parents; it builds on developing effective reappraisal strategies in the form of rational cognitions, known as protective factors against psychopathology. It seems that the ABM procedures focused on positive attention deployment emotion-regulation can be next incorporated in this program, given that it is a short intervention, which in newly developed game interface can be enjoyed by both parents and their children, with potential positive benefits for parental skills and parent–child relationships. Moreover, future studies should test whether involving children in the program, for playing an attention training game, would bring benefits in terms of its efficacy for child outcomes.

To sum up, we aimed to test an implicit parenting intervention for tracking positive responses in children is taking a step further explicit strategies used in parenting interventions for enhancing the positive facets of parent–child relationship. Our findings are in line with novel lines of research in the clinical field, providing initial support for the positive effects of implicit attention deployment and reappraisal-based emotion-regulation strategies used within online parenting programs. Future studies should focus on integrating innovative tools for improving emotion-regulation strategies in parents within online parenting programs and test their cumulative efficacy. Positive results could offer short enhancements to current evidence-based parenting programs with great benefits for children.

## Author Contributions

OD designed the study, the online platform, analyzed the data, and wrote the manuscript; DC and AJ contributed to the implementation of the protocol and data collection.

## Conflict of Interest Statement

The authors declare that the research was conducted in the absence of any commercial or financial relationships that could be construed as a potential conflict of interest.
